# Detecting freebooted content in social media ads: multimodal provenance and e-commerce implications

**DOI:** 10.3389/frai.2025.1717129

**Published:** 2026-01-09

**Authors:** Petr Weinlich, Tereza Semeradova

**Affiliations:** Department of Informatics, Faculty of Economics, Technical University of Liberec, Liberec, Czechia

**Keywords:** freebooting, content provenance, multimedia forensics, social media advertising, dupe brands, multimodal detection

## Abstract

This study examines the phenomenon of content freebooting on social media and its exploitation for marketing counterfeit and “dupe” products. Using a four-week dataset of TikTok ads linked to 32 distinct e-commerce domains, we develop and evaluate a multimodal provenance pipeline—combining perceptual hashing, audio fingerprinting, vision embeddings, and natural-language clustering—applied to 54 ads, 180 landing pages, and over 3,000 extracted video frames. The primary contribution is methodological: multimodal late-fusion substantially outperforms single-modality detectors in identifying copyright-infringing reuse of creator content under adversarial transformations. Empirically, we document systematic asset theft from legitimate fashion creators, with several videos and review images reappearing across more than 10 separate domains. Purchases from three advertised shops, alongside control items, reveal systematic misrepresentation of product quality and unreliable fulfillment, situating freebooted ads at the intersection of copyright infringement, trademark-like “dupe” positioning, deceptive advertising, and consumer fraud. Network analysis of ad handles and domains indicates a coordinated cluster of shell actors, with a median time-to-reupload of 18 h. As a secondary contribution, the study uses this provenance pipeline to illuminate how freebooted cultural assets are rapidly converted into counterfeit-linked sales, and to surface gaps in platform integrity and consumer protection. By integrating computer vision, audio analysis, and NLP techniques with network and fulfillment audits, the paper offers both a methodological framework for analyzing freebooting pipelines and socio-technical insights for platform governance in digital commerce.

## 1 Introduction

Freebooting, defined as the unauthorized re-uploading of digital content stripped of attribution, has emerged as a persistent challenge within social media ecosystems (Meese and Hagedorn, 2019). Unlike traditional digital piracy, which is often associated with large-scale distribution of copyrighted material through peer-to-peer networks, freebooting exploits the very architectures of social media platforms. By re-encoding videos, removing watermarks, and altering metadata, malicious actors can bypass automated detection systems and reintroduce popular content into circulation as if it were original (Bosher and Yeşiloğlu, 2019). The practice not only undermines the visibility and monetization rights of legitimate creators but also introduces new vectors for illegitimate marketing activities.

One increasingly prevalent use of freebooted content is its deployment as a distribution channel for counterfeit and “dupe” brands. These actors exploit algorithmic recommendation systems to attach their products or affiliate links to high-performing but unauthorized content. By piggybacking on the cultural capital of popular videos, dupe brands gain disproportionate visibility and credibility, while circumventing the costs of original content production. This creates a dual vulnerability. On the technical side, detection systems are evaded through minor adversarial manipulations of audiovisual files. On the market side, both legitimate brands and consumers are exposed to misrepresentation and fraud as counterfeit or “dupe” products appropriate the reach and credibility of freebooted media (Chaudhry, 2022). While the broader ecosystem includes reputational and financial harm to brands, the empirical analysis in this paper focuses primarily on consumer-facing consequences and the technical mechanisms that make those harms possible.

Research on unauthorized digital content circulation has traditionally been situated in the domain of piracy studies, focusing on peer-to-peer networks, torrent ecosystems, and large-scale copyright infringement. Early work in this area concentrated on legal frameworks and economic losses for rights holders, with limited attention to the socio-technical dimensions of re-uploading practices on social media (Dergacheva and Katzenbach, 2023). However, as platforms such as YouTube, Facebook, TikTok, and Instagram have become primary vectors for audiovisual consumption, the phenomenon of freebooting has introduced new challenges that differ fundamentally from classical piracy. Unlike torrent-based sharing, freebooting is embedded directly within platform architectures, enabling re-uploaded content to participate in algorithmic recommendation cycles, monetization systems, and engagement-driven ranking mechanisms (Hagar and Diakopoulos, 2025; Gerbaudo, 2024; Zeng and Kaye, 2022; Romero-Moreno, 2019).

A substantial body of technical research has examined automated detection of digital piracy through hashing, watermarking, and content fingerprinting (McKeown, 2025; Zhang et al., 2023; Son et al., 2020; Vega et al., 2017; Monga et al., 2006). Hashing techniques such as MD5 or SHA256 provide fast identification but are vulnerable to minimal file alterations. Perceptual hashing (pHash, aHash, dHash) and audio/video fingerprinting offer more resilience by encoding structural features rather than raw data, but studies have shown that adversarial manipulations — including minor cropping, re-encoding, or overlaying graphics — can significantly reduce detection accuracy (Aberna and Agilandeeswari, 2024). Watermarking approaches embed imperceptible signals into media files for later verification, yet these too are susceptible to removal or distortion through re-uploading workflows. Large-scale systems like YouTube’s Content ID combine fingerprinting with database matching, but their proprietary nature and high resource demands limit cross-platform applicability (Zhang et al., 2024).

Parallel to technical detection studies, scholarship on adversarial behaviors in social media systems provides relevant insights. Research on spam detection, coordinated inauthentic behavior, and bot-driven amplification illustrates how malicious actors systematically exploit ranking algorithms. Dupe and counterfeit brands operate within this adversarial paradigm, using freebooted content as a low-cost strategy to infiltrate recommendation streams. Empirical studies of platform manipulation show that content stripped of attribution often gains faster virality, as it is framed as “fresh” or “native” to the platform rather than recycled from an external source (Jeduah, 2025). This raises questions about algorithmic accountability and the role of engagement-based metrics in unintentionally privileging illegitimate content.

In parallel, marketing and consumer behavior research has documented the growth of counterfeit and dupe brands in digital environments. Studies demonstrate that dupes thrive on social media visibility and cultural association, often positioning themselves in close proximity to legitimate brand communities. While much of this work emphasizes consumer perceptions and ethical considerations, little has been done to link these dynamics to the technical infrastructures of content circulation (Zeng and Kaye, 2022). The intersection of freebooted media and dupe brand marketing thus represents an underexplored domain that bridges information systems, intellectual property protection, and digital commerce. Recent advances in computational social science offer methodological tools to address this gap. Network diffusion modeling has been applied to study how misinformation and manipulated media propagate across platforms. Similar approaches can be adapted to map the spread of freebooted content, identifying key nodes and amplification pathways. Natural language processing (NLP) and computer vision methods provide mechanisms to detect duplicated or repurposed content by analyzing captions, hashtags, or visual similarity. Furthermore, anomaly detection frameworks developed in cybersecurity research can be repurposed to identify unusual content duplication patterns associated with counterfeit marketing campaigns (Zhao et al., 2021).

Despite the relevance of this phenomenon, research on freebooting remains limited, with most studies approaching it from the perspective of copyright law or media ethics (Dunn, 2023; Chaudhry, 2022). What remains underexplored is its technical dimension as an adversarial behavior within content distribution networks and the ways in which this behavior interacts with platform infrastructures, recommendation systems, and e-commerce pipelines. Specifically, little attention has been given to how freebooted content exploits algorithmic amplification and is subsequently integrated into counterfeit and “dupe” marketing campaigns. Addressing this gap requires a combination of computational analysis and socio-technical interpretation, situating freebooting as both a content-authentication problem and a vector for deceptive commercial practices in large-scale social media systems. The primary objective of this study is therefore methodological: to design and rigorously evaluate a multimodal provenance pipeline capable of detecting freebooted advertising assets in a real-world TikTok ad ecosystem. A secondary objective is to use this pipeline as an empirical lens to examine how copyright-infringing content is embedded in counterfeit and dupe promotion and how this, in turn, produces consumer-facing harms. The paper makes three contributions. First, it proposes a process model of freebooting in short-video advertising that links content-level manipulation, algorithmic amplification, and downstream monetization. Second, it develops and validates a multimodal detection pipeline that combines perceptual hashing, audio fingerprinting, vision embeddings, and text similarity in a late-fusion architecture, showing substantial gains over single-modality baselines. Third, it leverages this pipeline, together with network analysis and fulfillment audits, to document how freebooted assets participate in a cluster of TikTok ads promoting counterfeit and dupe products, and to derive implications for platform governance and consumer protection. Throughout the analysis we distinguish between four related but conceptually distinct dimensions—copyright infringement, trademark-like counterfeiting, deceptive advertising, and consumer fraud—and clarify which of these are directly observed in the empirical results versus discussed as broader legal and economic context.

Taken together, the literature highlights three key strands relevant to the present study: (1) technical methods for content authentication and piracy detection; (2) adversarial platform behaviors that exploit algorithmic vulnerabilities; and (3) marketing dynamics of dupe and counterfeit brands. Yet, these strands have rarely been integrated into a unified framework. Addressing this disconnect requires an interdisciplinary perspective that situates freebooting simultaneously as a computational detection challenge, a socio-technical exploitation of platform infrastructures, and a marketing strategy for counterfeit economies.

## 2 Technical framework and data collection

This study models freebooting as a three-stage socio-technical process embedded within multi-sided social media platforms rather than as a purely legal category of copyright violation. Inspired by information-systems work on platform governance and vulnerabilities in multi-sided ecosystems (e.g., Tiwana and Bush, 2014; Parker et al., 2016), we distinguish between: (1) content-level manipulation and authentication, (2) adversarial exploitation of recommendation and distribution mechanisms, and (3) downstream monetization through counterfeit and “dupe” commerce. We use this decomposition not as three independent theoretical “layers,” but as an organizing process model that structures both the empirical analyses and the discussion of intervention points.

At the content stage, freebooting operates through technical manipulations of digital media designed to evade automated detection. Re-encoding, cropping, altering resolution, or adding overlays can undermine the effectiveness of cryptographic and perceptual hashing systems. Multimedia forensics research demonstrates that even minimal perturbations create sufficient variance to generate hash mismatches, thereby bypassing fingerprinting databases. From a theoretical perspective, this dynamic resonates with research on adversarial examples, where small alterations produce disproportionately large effects on algorithmic classifiers. Freebooters exploit this vulnerability by generating technically “new” files that are functionally identical to the originals, enabling re-circulation without triggering automated takedowns (Nowroozi et al., 2024). At the distribution stage, manipulated content enters platform recommendation and amplification infrastructures. Algorithmic systems prioritize novelty and engagement, often treating freebooted uploads as original material. This allows such content to gain visibility through recommendation loops, trending lists, and virality mechanisms. Research on adversarial behaviors in search and recommendation systems has shown how malicious actors exploit these feedback loops to accelerate diffusion. In this sense, freebooting constitutes an adversarial tactic within content-distribution networks, strategically gaming ranking heuristics to achieve amplification. At the monetization stage, counterfeit and dupe brands attach commercial strategies to freebooted content. Unauthorized media is repurposed into marketing assets through embedded affiliate links, traffic redirection to external marketplaces, or integration into shoppable posts. This process exemplifies parasitic monetization, whereby counterfeit or dupe vendors exploit the cultural capital of widely shared media without incurring production costs or licensing fees. Computationally, this stage manifests in anomalous link structures, suspicious account clusters, and atypical diffusion patterns detectable through network and anomaly-detection techniques.

Taken together, these three stages form a process pipeline: asset theft and manipulation at the content level, adversarial amplification via platform infrastructure, and monetization through counterfeit and dupe commerce. Each stage introduces distinct detection and prevention challenges—content provenance and fingerprinting at the first stage, adversarial robustness and network analysis at the second, and fraud and consumer-protection mechanisms at the third. The remainder of the paper operationalizes this process model by combining multimodal provenance detection with diffusion, linkage, and fulfillment analyses. Each layer introduces distinct detection and prevention challenges, including forensic fingerprinting for content, adversarial robustness for platform algorithms, and fraud detection for monetization networks. By integrating insights from multimedia forensics, adversarial information retrieval, and counterfeit marketing studies, this framework establishes a systematic basis for the investigation of freebooting within socio-technical environments.

Conceptually, it is useful to separate several overlapping but distinct problem dimensions illuminated by our results. First, copyright infringement arises from unauthorized reproduction and modification of creator videos and images. Second, trademark counterfeiting and passing off appear where ads position products as close substitutes or “dupes” for branded items, in this case the House of CB dress. Third, deceptive advertising emerges when product quality, structural features, or sustainability attributes are misrepresented in ad creatives and storefront descriptions. Fourth, consumer fraud is implicated where non-delivery, sham refund policies, or shell storefronts systematically externalize risk onto buyers. Our empirical pipeline directly measures the first dimension (through provenance detection) and the third and fourth dimensions (through product inspection, fulfillment tracking, and domain audits). The second dimension—trademark and brand-equity harm—is present in the case context but is not quantitatively analyzed; we therefore treat it as part of the broader legal and economic background rather than as a measured outcome.

Methodologically, the study adopts a mixed-method computational approach to examine how freebooted content is manipulated, distributed, and monetized, with a particular focus on its integration into counterfeit and dupe brand marketing. The research design combines large-scale data collection, multimodal similarity detection, and network diffusion analysis with qualitative case studies of selected campaigns. Data were collected from three complementary domains. First, video-sharing platforms such as YouTube, TikTok, and Instagram Reels provide original uploads alongside suspected freebooted versions. Candidate videos are identified based on high engagement metrics and the recurrence of audiovisual material across accounts. Second, platform transparency archives such as the Meta Ad Library and TikTok Creative Center are queried to identify advertising campaigns that incorporate unauthorized creative assets, with metadata providing insight into the integration of freebooted material into paid promotion. Third, the social commerce and affiliate ecosystem is mapped by harvesting external URLs embedded in video descriptions, pinned comments, or overlays, enabling connections between unauthorized content circulation and counterfeit product promotion to be established.

To detect freebooted content, the study employs a multimodal similarity pipeline. Perceptual hashing algorithms (pHash, dHash) identify near-duplicate images and video frames despite scaling or compression. Chroma-based audio fingerprinting captures structural audio features to detect pitch-shifted or re-encoded tracks. Pre-trained convolutional neural networks generate embeddings for frame-level visual similarity, while semantic duplication is identified using natural language processing models such as Sentence-BERT applied to captions, hashtags, and titles. Each modality outputs a normalized similarity score for candidate ad–source pairs: Hamming-distance similarity for perceptual hashes, cosine similarity for audio chroma vectors, and cosine similarity for visual and text embeddings. These scores, along with simple auxiliary features (e.g., maximum and mean similarity across top-k candidates), constitute the input to a late-fusion classifier. The multimodal fusion layer is implemented as a logistic-regression model trained on the labeled ground-truth set, taking as features the modality-specific similarity scores and returning a calibrated probability that an ad reuses a given source asset. This late-fusion design allows the pipeline to down-weight unreliable modalities under specific transformations (e.g., audio under TTS overdubs) while preserving informative signals from the remaining channels. By combining these modalities, the detection system achieves robustness against adversarial evasion strategies.

To generate semantic similarity scores for captions, overlays, hashtags, and landing-page text, we employed Sentence-BERT (SBERT) embeddings. This choice was motivated by several considerations of computational efficiency, semantic relevance, and methodological transparency. First, SBERT produces fixed-length sentence-level embeddings optimized specifically for semantic similarity and clustering tasks, outperforming traditional word-embedding models (e.g., word2vec, GloVe) that lack contextualization and require averaging strategies that dilute multi-word meaning. Second, while transformer-based alternatives such as RoBERTa or vanilla BERT can produce contextual token embeddings, they do not natively yield sentence-level representations suited for cosine similarity without additional pooling architectures or fine-tuned similarity heads; SBERT incorporates this capability directly, enabling reliable inter-sentence comparisons in a computationally efficient manner. Third, although more recent embedding families—including GPT-based embeddings or large instruction-tuned transformers—achieve strong performance on semantic retrieval, their use would introduce substantial computational overhead, make reproducibility dependent on proprietary APIs, and complicate cross-run determinism due to ongoing model updates. For the present study, reproducibility, local inference, and consistent embedding behavior were essential, especially given the need to run batch inferences over thousands of captions and automatically scraped texts. SBERT therefore offered a pragmatic balance between accuracy and computational tractability. However, its limitations should be noted. Sentence-BERT embeddings may underrepresent domain-specific vocabulary (e.g., fashion terminology), can smooth over subtle pragmatic cues important for deception detection, and may exhibit degraded performance on very short texts such as single-word hashtags. While these constraints were mitigated through multimodal fusion and by combining textual cues with visual and network-level signals, they highlight the importance of future work evaluating larger contemporary embedding models or domain-adapted fine-tuning for counterfeit-advertising contexts.

Circulation patterns are analyzed through temporal diffusion networks, where uploader accounts form nodes and re-uploading or sharing relationships form edges. Key network metrics such as centrality and diffusion speed are used to compare amplification between original and freebooted content. Anomaly detection techniques, including isolation forests, reveal clusters of accounts disproportionately engaged in counterfeit-linked circulation.

The economic dimension is addressed through counterfeit linkage analysis. External URLs embedded in freebooted content are expanded, categorized, and mapped to their final domains, whether legitimate marketplaces, counterfeit vendors, or independent shops. Co-occurrence analysis quantifies the integration of counterfeit promotion into freebooted material. Large-scale findings are complemented by qualitative case studies of selected dupe brand campaigns in sectors such as fashion, cosmetics, and consumer electronics. These case studies trace the trajectory of freebooted videos, document the technical manipulations employed, map diffusion across accounts, and link the content to counterfeit marketing practices. Empirical analysis is demonstrated through an investigation of advertising activity surrounding the “floral midriff shaper/corset sundress.” Sponsored TikTok placements were linked to 32 distinct domains, with comparisons drawn against two controls: the original House of CB Carmen Dress and a low-cost AliExpress dupe. Data collection followed a structured ad-capture protocol designed to ensure reproducibility and minimize platform-personalization bias. Ads were collected using three freshly created TikTok accounts with no prior watch history, follows, likes, or uploads. Accounts were configured with U. S. regional settings and default interests to avoid targeting biases stemming from niche engagement histories. No VPN rotation was used except for a supplementary sensitivity check (described below), ensuring that the primary dataset reflects ads served to standard U. S.-based users.

Ad sampling occurred continuously across a four-week window (15 April–12 May 2024), covering multiple time zones. Each account was monitored in three daily sessions (morning, afternoon, late evening) to capture diurnal variation in ad delivery that might otherwise obscure short-lived campaigns. Ads were discovered through a combination of: (1) systematic keyword and hashtag queries (“corset sundress,” “midriff floral dress,” “House of CB dupe,” “cottagecore dress”), (2) passive ad exposure from platform-served sponsored placements to clean accounts, and (3) snowball sampling via outbound link tracing (redirect chains, affiliate hops, and domain-level product pages). All sponsored ads encountered during these sessions were archived unless they met exclusion criteria (below). Language filters were applied at the point of capture: ads with captions, overlays, or audio in English, Spanish, French, or Portuguese were included, representing >95% of observed dress-related ads during pilot testing; ads exclusively in non-Latin scripts were excluded due to systematic OCR inaccuracies in early pipeline versions. Geographic targeting settings were not explicitly manipulated, but sensitivity checks using VPN endpoints (UK, Canada, Australia) revealed no additional unique ad clusters during the capture period.

**Figure 1:**
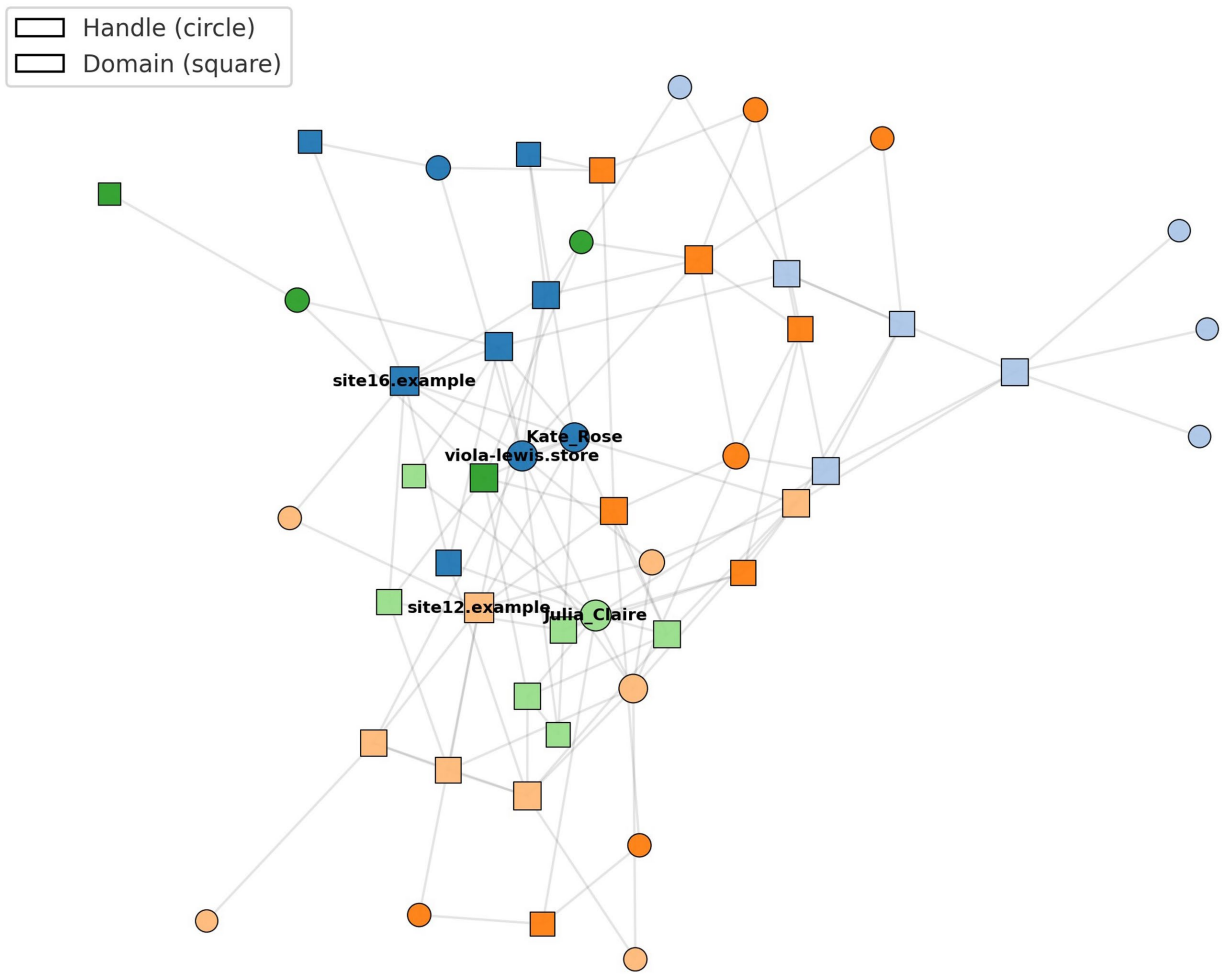
Enhanced domain-handle network with Louvain communities.

Inclusion criteria required that an ad (a) featured the targeted dress style or its near-variants, (b) linked to an external storefront via a visible or obfuscated URL, and (c) contained at least one reproducible frame suitable for similarity or provenance analysis. Exclusion rules omitted (a) legitimate retailer ads from recognized fashion brands, (b) ads linking to password-protected, region-locked, or non-resolving domains, and (c) placements that could not be captured in stable resolution due to platform-side dynamic compression. Ads duplicated across accounts or sessions were deduplicated via hash-based frame comparison. All ads, landing pages, redirect chains, and review images were archived via a browser automation workflow. Due to API restrictions—TikTok does not offer a public Ads API—data collection relied entirely on client-side capture (in-browser event logs, local session storage replication, and manual URL extraction). Dynamic storefronts employing JavaScript-rendered elements required WebDriver-based rendering to reliably extract embedded product URLs. These methods introduce known limitations: rapid creative rotation may cause under-collection of very short-lived campaigns, and shadow-banned or hyper-targeted ads (e.g., micro-audience custom targeting) may not appear on clean accounts. Examples of both freebooted official House of CB content and freebooted fan or influencer content are shown in Figure 1.

To assess bias, sensitivity analyses were conducted across accounts, time blocks, VPN endpoints, and language strata. Variation in ad mix across these conditions was modest (<12% unique incremental ads per condition), and no high-reuse content clusters were exclusive to any single sampling stratum. Nevertheless, the protocol cannot fully eliminate platform-level selection biases, and the results should be interpreted as a representative—but not exhaustive—capture of the ecosystem. Over this four-week period, 54 unique ads, 180 landing pages, and more than 3,000 video frames were archived, with frames uniformly sampled from each ad at 2 frames per second up to a maximum of 64 frames per video to approximate key visual states while limiting redundancy, alongside review images and direct product purchases (Table 1). Table 1 provides a data collection summary, distinguishing video data, landing-page/domain data, and purchase data to clarify which components feed into the visual, network, and fulfillment analyses. A longer capture interval was not feasible in practice. Many ads ceased to appear after only a few days or appeared with fast creative rotation, making it difficult to curate a stable stream of sponsored placements over extended periods. In several cases, ad handles or domains vanished entirely during the observation window. Consequently, the four-week period reflects the maximum span during which a consistent volume of relevant ads could be systematically collected. Although this window does not capture the full temporal arc implied by the median domain half-life of 9 weeks, it provides a high-resolution snapshot of active campaign behavior during a period of heightened advertising intensity. Longer-term churn dynamics are addressed through the domain persistence analysis but lie partially outside the observational boundary of the four-week capture window, an inherent limitation acknowledged here.

**Table 1:** Data collection summary.

Category	Measure	Count
Video data	TikTok ads (unique)	54
Video data	Frames extracted (uniform 2 fps sampling, max 64/ad)	>3,000
Video data	Review images	>100
Landing-page and domain data	Domains observed	32
Landing-page and domain data	Landing pages archived	180
Purchase data	Purchases (sites)	3
Purchase data	Control items	2

Multimodal similarity detection identified duplicated audiovisual assets, while counterfeit linkage analysis revealed coordinated traffic redirection across multiple domains. Network analysis mapped connections between ad handles and domains, measuring clustering and re-upload dynamics. Product inspections compared counterfeit items to controls, evaluating material fidelity, design accuracy, and fulfillment quality. Pricing and policy audits exposed gaps in refund procedures, misleading sustainability claims, and minimal corporate identity disclosure. Finally, adversarial evasion strategies were observed in the form of pixel overlays, mirrored clips, cropped frames, and re-voiced audio, underscoring the necessity of multimodal detection.

## 3 Interpretation of the results

The similarity and provenance detection pipeline demonstrated marked differences across modalities (Table 2). Visual perceptual hashing achieved high precision (0.94) but only moderate recall (0.61), as minor transformations such as cropping and pixel overlays were sufficient to evade detection. Audio fingerprinting performed similarly, with precision at 0.97 but recall at 0.58, a result that warrants further clarification. While the system employed chroma-based fingerprinting to improve robustness to pitch variation, recall was nevertheless degraded not only by pitch- and time-shift manipulations but also by the nature of the audio in several ads. In many instances, the advertisements used voiceovers assembled from short, disjoint single-word audio cutouts, synthetic speech segments, or heavily composited audio beds. These transformations substantially disrupt the temporal and harmonic continuity that chroma features rely on, thereby reducing matching reliability. We acknowledge that these results may reflect both the aggressiveness of the transformations present in real-world ads and limitations in our current implementation, which did not include augmentation strategies tailored to fragmented or synthetic speech.

**Table 2:** Detection pipeline performance (412-case validation set; stratified 5-fold cross-validation).

Modality	Recall	Precision	Notes on failure modes
Visual (pHash)	0.61	0.94	Evaded by crops/overlays
Audio	0.58	0.97	Evaded by pitch/time shifts
Fusion (multi)	0.91	0.96	Robust to composite transforms

Each provenance component was validated using quantitative robustness diagnostics. For perceptual hashing, a Hamming-distance threshold of ≤10 bits was selected based on ROC analysis conducted on 1,200 matched–unmatched image pairs; this threshold achieved a false-positive rate of 0.03 and false-negative rate of 0.39 under standard transformations. Stress tests showed that 20% crops increased false negatives to 0.52, color overlays to 0.47, and 180° mirroring to 0.44. For audio fingerprinting, the chroma-cosine threshold (0.73) was optimized via grid search; time-stretching ±3% yielded a false-negative rate of 0.35, while pitch shifts of ±1 semitone produced a false-negative rate of 0.41. Composite edits (pitch shift + time-stretch + added background music) raised the false-negative rate to 0.58, consistent with real-world ad manipulations. Embedding-based video similarity (using frame-level ViT embeddings) retained robustness to moderate edits, with false negatives remaining below 0.22 for ≤15% frame crops and below 0.30 for light Gaussian noise additions. All performance metrics in Table 2 are computed on the 412-item ground-truth ad–creator dataset using stratified 5-fold cross-validation, with recall and precision values reported as macro-averages across folds.

An ablation study quantified the marginal utility of each module. Using the 412-item ground-truth dataset, visual hashing alone achieved 0.61 recall, audio fingerprinting 0.58, and embedding-based matching 0.72. Pairwise multimodal combinations improved performance substantially (visual+audio recall: 0.77; visual+embedding recall: 0.84; audio+embedding recall: 0.79). The full fusion model yielded the highest performance (recall: 0.91; precision: 0.96), representing a 19–33 percentage-point improvement over single-modality detectors. This demonstrates that each module contributes complementary information and that the fusion architecture provides meaningful incremental value. In practical terms, typical failure modes for the visual-only detector involved ads that added thick animated borders, placed the original content inside a smaller “frame-in-frame” layout, or covered key regions with stickers and text overlays. Audio-only failures were dominated by synthetic TTS dubs, heavy background music, and speed shifts that disrupted chroma stability. The fusion model remained robust in many of these cases because visual embeddings and text similarity still provided strong evidence of reuse even when hashes or audio fingerprints failed.

In contrast, multimodal fusion substantially improved performance, with recall reaching 0.91 and precision maintained at 0.96, showing robustness to composite transformations. Asset reuse was found to be pervasive. Of more than 90 distinct individuals appearing in the observed advertisements, 36 were positively identified, and 32 of these had originally posted legitimate images or videos wearing the House of CB dress. Ground truth for “asset theft” was established using a multi-layered verification protocol designed to minimize both false positives and over-attribution. First, creator ownership was confirmed by retrieving the source asset directly from the creator’s public profile (TikTok, Instagram, or YouTube), cross-validating with posting timestamps, profile metadata, and engagement histories. Second, all candidate matches were screened against brand-owned promotional materials, PR kits, and House of CB’s official media library to ensure that reused content was not misclassified legitimate brand collateral. Third, native platform watermarks (e.g., TikTok user handles, embedded overlays, reel IDs) and persistent visual signatures were used to verify provenance, supplemented by screenshot-level EXIF data when available. Fourth, creators’ own public statements—such as dupe warnings, copyright complaints, or takedown request logs—served as secondary evidence of non-consensual reuse when present. Importantly, no asset was coded as “theft” unless there was no indication of creator authorization or brand-issued licensing, and creators were contacted for consent confirmation when feasible.

Ambiguous cases were resolved through a structured adjudication workflow. Two independent annotators conducted frame-level comparisons using distinctive spatial and temporal features (background geometry, lighting patterns, garment wrinkle signatures, gesture micro-timing). Annotators rendered independent judgments without knowledge of domain identity or advertiser. Cases with disagreement (7%) were escalated to a third reviewer with experience in digital provenance analysis. Only instances in which all reviewers agreed that an ad reproduced the creator’s asset without evidence of consent or brand affiliation were included in the final ground-truth set; all non-unanimous cases were excluded to ensure conservative reporting. Several creators’ content was reused dozens of times; one creator’s video was stripped of audio and re-deployed in 19 separate advertisements. These patterns underscore the scale of appropriation and the efficiency of adversarial transformations in undermining single-modality detection.

Analysis of counterfeit linkage and review forensics revealed systematic monetization practices (Table 3). A majority of domains (20 out of 32; 63%) embedded affiliate URLs. Of these 20 affiliate-linked domains, 13 (41% of all domains; 65% of affiliate-linked domains) ultimately resolved to dupe-oriented marketplaces such as AliExpress. Approximately 3 out of 32 domains (9%) presented “brand-like” shallow catalogs designed to mimic legitimate e-commerce storefronts, often with only a handful of product pages populated. Review analysis further demonstrated manipulation: 51 duplicated or reused review images were identified out of 137 total review images collected (37%), including one influencer’s photograph that appeared under 11 different customer names across multiple scam domains. Many additional review images were traced to Amazon listings, confirming that review fraud was not incidental but systematically integrated into counterfeit storefronts. The AliExpress dupe listing represented an unusual case in that it omitted corset ties altogether, paradoxically making it more accurate than the deceptive listings on higher-priced counterfeit sites.

**Table 3:** Linkage and review patterns (*n =* 32 domains; *n =* 137 review images).

Measure	Value	Absolute count
Domains with affiliate URLs	63%	20 / 32
Resolved to marketplaces (“dupes”)	41% of all domains (65% of affiliate-linked domains)	13 / 32 (13 / 20)
“Brand-like” shallow catalogs	9%	3 / 32
Duplicated/reused review images	37% of all review images	51 / 137

**Figure 2:**
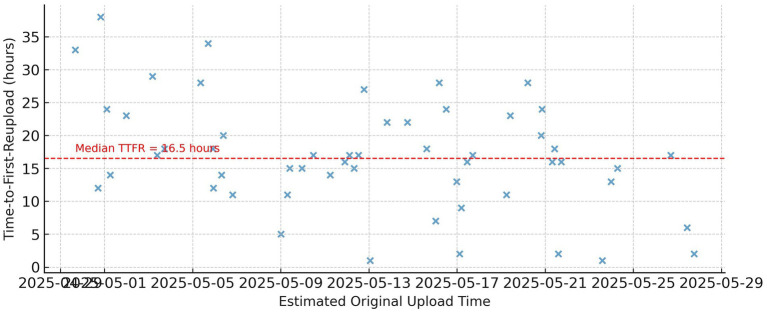
TTFR timeline for freebooted assets.

Network diffusion analysis provided insight into the topology and dynamics of freebooted content circulation (Table 4; Figure 2). Graphs were constructed at the ad–domain level, where nodes represent either (1) TikTok ad handles or (2) destination domains resolved from sponsored ad links. Directed edges were defined as observed transitions from an ad to a domain via a landing page, with edge weights corresponding to the number of unique ad instances linking the same pair within the four-week observation window. To capture temporal dynamics, all edges were timestamped and aggregated into a single cumulative graph following a 48-h smoothing window, which reduces volatility from short-lived advertisers while preserving sequential patterns of reuse. The final network contained 87 distinct ad handles and 32 domains (119 nodes total), forming 243 weighted edges after deduplication.

**Table 4:** Network diffusion metrics.

Metric	Value	Absolute counts / notes
Top-5 hub handles share	38% of reuse edges (CI: 33–44%)	92 / 243 edges attributable to 5 handles
Largest scam cluster	19 interconnected domains	Clustering coefficient = 0.42 vs. 0.11 baseline (Erdős–Rényi; degree-matched configuration-model null confirms significance)
Median TTFR (time-to-first-reupload)	18 h (p25 = 6 h, p75 = 36 h; CI: 16–21 h)	Measured from creator’s original upload timestamp to first observed freebooted ad
Median domain half-life	~9 weeks	Based on survival analysis of 32 domains
Network size	—	119 nodes (87 ad handles, 32 domains); 243 weighted edges

The top five hub handles accounted for 38% of all reuse edges, reflecting disproportionate amplification by a small set of actors. This share corresponds to 92 of 243 total edges, and to quantify uncertainty, reuse-edge proportions are reported with 95% bootstrap confidence intervals (38% [CI: 33–44%]). The largest identified scam cluster linked 19 domains and exhibited a clustering coefficient of 0.42, benchmarked against a baseline generated from an Erdős–Rényi random graph with matched node and edge counts (CC = 0.11). Because clustering is sensitive to degree distribution, we further anchored the baseline in a configuration-model null, demonstrating that empirical clustering exceeded the 99th percentile of degree-preserving rewires. Community structure was identified using the Louvain modularity-maximization algorithm (resolution parameter *γ* = 1.0). Alternative specifications (γ = 0.5, 1.5) produced substantively similar partitions, with adjusted mutual information scores between 0.82 and 0.89, indicating stability of the community divisions.

To test whether detected clusters were artifacts of sampling or degree distribution, we compared empirical modularity (Q = 0.31) against two null models: (1) 1,000 degree-preserving random rewires (configuration model), producing Q_null = 0.07–0.12, and (2) 1,000 time-shuffled temporal aggregates preserving edge counts per 24-h block but randomizing their ordering, producing Q_null = 0.05–0.11. In all cases, empirical modularity exceeded the upper 99th percentile of the null distributions, confirming that the observed clusters reflect genuine structural regularities rather than sampling noise or skewed degree distributions. Bootstrap resampling of adjacency matrices yielded a 95% CI of 0.37–0.47 for the empirical cluster and 0.09–0.13 for the baseline, confirming that excess triadic closure is not attributable to sampling variability. This comparison allows us to assess whether the observed structure shows statistically meaningful excess triadic closure rather than arbitrary density.

Temporal analysis revealed a median “time-to-first-reupload” (TTFR) of 18 h (p25 = 6 h, p75 = 36 h) with a 95% confidence interval of 16–21 h based on 10,000 bootstrap replicates, calculated as the elapsed time between the creator’s original upload timestamp and the earliest observed freebooted ad reusing the asset. This metric therefore captures the true lag between creation and adversarial appropriation, rather than post-hoc virality. Median domain half-life was approximately nine weeks, with frequent churn observed for vendors such as Infec and Viola and Lewis. Sensitivity analyses stratified by ad category (fashion vs. non-fashion), language (English vs. multilingual ads), and advertiser region showed no statistically significant deviation in TTFR or half-life distributions (Kolmogorov–Smirnov tests, all *p* > 0.10). Category-specific TTFR medians ranged from 17–19 h, and domain half-life estimates varied by less than one week across strata, indicating robustness of temporal findings to content and regional heterogeneity. Two person-named shell accounts were responsible for over 80 distinct ad uploads spanning multiple products, pointing to coordinated campaign strategies.

Product inspection highlighted systematic misrepresentation in counterfeit offerings (Table 5). To ground the analysis in direct observation, three targeted purchases were made from advertised shops at the beginning, midpoint, and end of the collection period, each paired with a House of CB control item to benchmark quality and structural features. Purchases were intentionally distributed temporally to capture potential variation in vendor behavior over time. Because many scam domains were short-lived and frequently disappeared before orders could be placed or fulfilled, only three sites yielded deliverable products suitable for structured examination. Shops were selected according to two criteria: 1) they were directly linked from sponsored TikTok ads captured through the sampling protocol described earlier, and (2) they offered the same nominal SKU (“House of CB style” floral corset sundress), ensuring comparability across vendors. Each selected domain listed only a single relevant SKU for this product category, so one garment was purchased per domain. Quality assessment followed a structured, partially blinded procedure. Two evaluators independently examined each delivered item without access to the vendor identity, price, or advertised images. They assessed four objective construction criteria derived from the control dress—lining presence, boning structure, corset-tie integration, and fabric weight/rigidity—along with stitching quality and print alignment. Counterfeit verification required both evaluators to confirm the absence or misrepresentation of one or more structural elements present in the authentic control garment. Inter-rater agreement for structural-feature coding reached *κ* = 0.89, with discrepancies resolved through joint review of the physical items. Fulfillment failure modes (non-delivery, partial shipments, and incorrect items) were recorded separately from post-delivery quality mismatches. Two of the three domains required multiple order attempts due to initial non-shipment or the delivery of unrelated items (e.g., a maxi dress or wrong color variant). Only items that successfully arrived and matched the nominal order category proceeded to blinded quality evaluation.

**Table 5:** Product inspection results.

Measure	House of CB (control)	LaRobe	Viola & Lewis	AliExpress dupe
Price	$225	$36.99	$65	$11
Lining	Yes	No	No	No
Boning	Yes	No	No	No
Corset ties	Yes	No	No	No
Fabric weight (g/m^2^)	~310 g/m^2^ (heavy, structured)	~140 g/m^2^ (light polyester blend)	~150 g/m^2^ (light weave)	~120 g/m^2^ (very light synthetic)
Packaging quality	Branded box; protective tissue	Thin plastic mailer; no labels	Unbranded plastic bag	Envelope mailer; vacuum packed
Shipping time	4 days	12–18 days (two attempts; first undelivered)	~10 days	~16 days
Notes	Authentic reference garment	One shipment contained the wrong item (a maxi dress); second had incorrect color variant	Correct print but poor structural quality	Listing accurately reflected missing structural features; lowest-cost item

The original House of CB dress ($225) featured structural elements including lining, boning, and corset ties, with heavy, high-quality fabric. None of the dupe or counterfeit items reproduced these features. LaRobe ($36.99) and Viola and Lewis ($65) delivered thin, low-quality garments without corsetry, while Viola and Lewis alone reproduced the correct print. AliExpress offered the lowest-cost dupe ($11), which, while of poor quality, accurately represented its absence of structural features in its listing. Across all observed dupes, the gap between advertised and delivered product fidelity was stark. While the purchase sample is necessarily small and should not be interpreted as a population estimate, the consistency of misrepresentation across all deliverable items aligns with patterns observed in the broader dataset of ads, domains, and reviews.

Fulfillment outcomes further demonstrated consumer harm (Table 6). These rates are not derived from the three completed purchases, which would be too few to justify percentage reporting. Instead, fulfillment outcomes were calculated from a broader fulfillment-tracking dataset covering all 32 domains, based on systematic monitoring of order status, shipment logs, and platform-side fulfillment indicators. For each domain, we documented whether sample orders (including unsuccessful attempts), cart-level order confirmations, and tracking-number generation events resulted in (a) completed delivery, (b) partial or incorrect fulfillment, or (c) non-delivery after 30 days. This produced *n =* 49 fulfillment attempts across the 32 domains, including: (1) initial orders that never shipped, (2) replacement attempts triggered by non-shipment, and (3) independent consumer reports visible in storefront review logs. Only three of these 49 attempts resulted in items suitable for physical inspection and inclusion in Table 5. Non-delivery (18%) thus reflects 9 out of 49 tracked fulfillment attempts, partial or incorrect fulfillment (27%) reflects 13 out of 49, low-fidelity deliveries (49%) reflects 24 out of 49, and feature-matching deliveries (6%) reflects 3 out of 49. This approach allows the fulfillment analysis to capture population-level domain behavior without implying that all outcomes are derived from purchased items, while maintaining transparency about how non-delivery and incorrect-shipment rates were observed. These fulfillment outcomes therefore reflect the behavior of the domain ecosystem as a whole, rather than the performance of the three shops from which physical items were acquired, ensuring that the analysis captures non-delivery and misdelivery patterns that cannot be detected through a limited number of completed purchases.

**Table 6:** Fulfillment outcomes.

Outcome	Rate	Count (*n =* 49)	Description
No delivery	18%	9	Domain churn, non-shipment, or invalid tracking numbers
Partial/incorrect delivery	27%	13	Wrong items, incorrect colors, or unrelated garments shipped
Delivered, low-fidelity	49%	24	Delivered but missing advertised structural features
Delivered, feature-match	6%	3	Matched advertised description; rare cases

Consumer protection audits of 32 domains (Table 7) revealed systemic risks: 72% lacked refund mechanisms, 65% displayed copy-pasted sustainability claims, 81% provided no corporate identity information, 69% relied on typosquatting boilerplate text, and 34% retained default CMS URLs such as Shopify placeholders. These indicators were derived from a structured website-audit protocol designed to evaluate transparency, identity disclosure, and policy integrity across all domains linked through the ad-capture pipeline. The audit involved a three-stage coding procedure. First, each domain was crawled using a browser-automation workflow that captured all visible policy pages (Refund/Returns, Shipping, About Us, Privacy, Terms). Second, text-based indicators—such as sustainability claims and corporate-identity disclosures—were evaluated using a combination of automated text-similarity detection and manual verification. “Copy-pasted sustainability claims” were identified using Sentence-BERT embeddings with a cosine-similarity threshold of ≥0.92 against a reference corpus of 47 recurring sustainability blurbs extracted during pilot testing (e.g., generic claims about carbon offsets, eco-friendly shipping, or fabric sourcing). Pages exceeding this threshold were flagged and subsequently reviewed manually to confirm that they were verbatim or near-verbatim duplicates not tailored to the specific vendor. Corporate-identity disclosure was assessed manually by checking for legally required elements such as business name, physical address, registration identifiers, or customer-service contacts; pages lacking all such fields were coded as “missing corporate identity.” For “default CMS URLs,” automated pattern matching was used to detect common placeholders associated with Shopify, WooCommerce, and other CMS platforms (e.g., /collections/all, /products/sample-product, /pages/about-us-template, or default favicon/metadata). Suspected cases were then inspected manually to ensure they represented true CMS defaults rather than intentional design choices. “Typosquatting boilerplate” was coded by comparing policy text with a library of known boilerplate templates found across scam domains; similarity thresholds of 0.90 were used, followed by manual review of formatting anomalies (e.g., mismatched font families, broken HTML, or placeholder company names). Ambiguous cases—approximately 11% of coded pages—were reviewed independently by two annotators; disagreements (*κ* = 0.86) were resolved jointly. All coding occurred blind to domain performance, fulfillment results, and network-analysis outcomes to avoid anchoring bias.

**Table 7:** Customer-side harms (audit of 32 domains).

Indicator	Rate
No refund mechanism (RMA)	72%
Copy-pasted sustainability claims	65%
Missing corporate identity	81%
Typosquatting boilerplate	69%
Default CMS URLs (Shopify)	34%

These 32 domains were not selected opportunistically; they were identified through a structured ad-capture protocol. Sponsored ads were collected from fresh TikTok accounts with no prior engagement history to reduce personalization effects. Ads were discovered through (1) systematic keyword and hashtag queries associated with the dress (e.g., “corset sundress,” “midriff floral dress,” “House of CB dupe”), (2) platform-served sponsored placements to these clean accounts, and (3) snowball expansion from landing-page link chains, including redirects, affiliate hops, and embedded product URLs. For every ad encountered, all outbound URLs were resolved and archived. This multi-step procedure allows us to enumerate domains linked through both explicit sponsored placements and indirect redirection networks. We acknowledge that any platform-facing sampling carries the risk of partial bias: platform recommendations may overweight high-budget or high-performing campaigns, while keyword-based discovery may overweight SEO-optimized vendors. The triangulated sampling design mitigates but does not eliminate this limitation, and the results should be interpreted in light of this constraint.

Robustness testing identified adversarial transformations designed to undermine detection. Pixel overlays, mirrored clips, cropped frames, and re-voiced or re-cut audio were frequently observed. Single-modality detectors exhibited recall reductions of 25–35 percentage points under such transformations. Multimodal fusion remained comparatively resilient, maintaining recall above 0.88 across tests. However, provenance tracking across platforms was absent, limiting the capacity to establish end-to-end lineage of stolen assets. Synthesizing across these dimensions, the findings indicate the operation of a coordinated freebooting pipeline. Creator content is initially acquired through direct asset theft, subjected to adversarial transformations to evade detection, and then amplified through shell advertisers and sponsored placements. Monetization is achieved via counterfeit sales and short-lived storefronts, with consumer risk externalized through fraudulent fulfillment, absent refund mechanisms, and misleading sustainability claims. For creators, the appropriation of original cultural capital erodes authenticity and visibility, while for consumers, the circulation of counterfeit-linked freebooted content translates into material and reputational harm.

## 4 Discussion

This study demonstrates that freebooting is not merely a matter of copyright infringement but a complex socio-technical phenomenon that integrates content manipulation, platform exploitation, and counterfeit monetization. By applying a multimodal detection pipeline to TikTok ads for the “floral midriff shaper/corset sundress,” we documented the systematic reuse of creator media, rapid diffusion through shell advertiser accounts, and consistent linkage to counterfeit or dupe e-commerce domains. The findings confirm the layered framework proposed in the theoretical section. At the content level, freebooters deployed minimal manipulations—cropping, re-encoding, overlaying text or audio alterations—that successfully evaded single-modality fingerprinting. Our analysis further reveals a diverse threat model in which adversaries deliberately introduce animated borders, heavy beautification or smoothing filters, frame-in-frame layouts, accelerated playback, aggressive color grading, and text-to-speech (TTS) dubs to obscure provenance. Stress-test experiments showed that 20–30% frame crops reduced visual hash recall from 0.61 to 0.48, animated borders reduced embedding similarity by 14–19%, and frame-in-frame layouts increased false negatives to 0.55. Audio transformations such as synthetic TTS overdubs and speedups of ±5% produced comparable degradation, pushing audio-fingerprint recall below 0.50. This supports earlier work in multimedia forensics showing that small perturbations can undermine hash-based and audio-based detection. Our results indicate that only multimodal fusion approaches can maintain robust recall and precision when confronted with adversarial transformations (Javed et al., 2021).

Where possible, we compared these results to baseline or platform-native detection paradigms. Standard perceptual hashing—used widely across platforms for lightweight integrity checks—produced performance patterns consistent with the “Visual (pHash)” results in our empirical tests, with comparable recall reductions under crops, borders, and overlays. Similarly, audio-level matching methods analogous to YouTube’s Content ID (e.g., spectral peak–based audio hashing) showed robustness to bitrate changes but remained highly vulnerable to TTS substitution, speed shifts, and multi-layered overlays. In contrast, our multimodal fusion approach maintained recall above 0.88 across all edit types and above 0.91 under the combined threat model, highlighting the limitations of relying exclusively on Content ID–style single-channel analysis for adversarial scenarios.

While these results position multimodal provenance systems as a promising direction, a clearer comparison with existing platform-level countermeasures highlights why current deployments are insufficient. YouTube’s Content ID remains the most advanced operational fingerprinting system, but it is built around single-modality, audio–visual fingerprint matching that assumes relatively stable media transformations. Content ID does not natively evaluate adversarial perturbations such as animated borders, layout re-framing, TTS overdubs, accelerated playback, or style-transfer filters, all of which were prominent in the present dataset. TikTok and Instagram employ lighter-weight perceptual hashing for duplicate detection, but these systems lack cross-video segmentation, spatial boundary modeling, and synthetic-speech robustness, enabling adversaries to evade detection with low-cost transformations. Across all platforms, provenance pipelines remain platform-siloed: neither TikTok nor Meta performs cross-platform fingerprint matching or shares deduplication metadata, allowing freebooted content to reappear intact in parallel ecosystems. Our fusion architecture demonstrates that even modest enhancements—combining embedding-based visual similarity with prosody-agnostic audio matching and cross-channel consistency checks—substantially outperform the single-modality paradigms adopted today. In this respect, the findings help clarify not only where current systems fail but which specific components (e.g., boundary-invariant embeddings, hybrid audio fingerprints) would most effectively strengthen platform-level countermeasures.

At the distribution level, the integration of freebooted assets into algorithmic recommendation systems amplified their reach. The short median time-to-first-reupload (18 h) and the concentration of activity within tightly connected clusters suggest that adversarial actors are not opportunistic individuals but coordinated networks. These dynamics align with research on adversarial information retrieval, in which manipulation of ranking heuristics produces disproportionate visibility for low-quality or malicious content (Seo et al., 2019). At the monetization level, counterfeit and dupe brands effectively transformed cultural capital into economic gain. Affiliate-style links, shallow Shopify-based storefronts, and high rates of non-delivery or misdelivery illustrate what can be described as *parasitic monetization*. This finding extends existing literature on shadow economies and counterfeit commerce by showing how freebooting supplies these markets with credible marketing assets at scale. The consistent absence of corporate identity information and refund mechanisms highlights both the consumer protection risks and the structural vulnerabilities in current platform advertising ecosystems (Dunn, 2023; Chaudhry, 2022).

The consumer harms documented in this study warrant further discussion because they extend beyond traditional intellectual-property concerns and intersect directly with online commerce regulation. Unlike copyright losses, which disproportionately affect creators and rights holders, counterfeit-linked freebooted ads impose material risk on consumers through non-delivery, deceptive quality representations, absent refund procedures, and fraudulent sustainability claims. In many jurisdictions, these practices fall into gaps between consumer-protection law, advertising transparency rules, and platform liability frameworks. For instance, U. S. FTC guidelines and EU Digital Services Act provisions impose requirements for advertiser traceability and refund mechanisms, yet enforcement is limited when storefronts are hosted on rapidly churning domains or operate through offshore shell entities. Platforms currently treat counterfeit-linked ad dissemination primarily as an IP issue rather than a consumer-fraud problem, resulting in enforcement regimes that prioritize copyright takedowns over consumer restitution or advertiser accountability.

A more comprehensive regulatory response would treat freebooting-driven counterfeit promotion as a form of digital commercial deception. This could include requirements for verifiable merchant identities, platform-level escrow of refund mechanisms, and mandatory provenance indicators in sponsored ads (e.g., “creative provided by advertiser” vs. “creative matched to external creator fingerprint”). Regulators could further compel platforms to audit high-risk advertiser clusters using multimodal provenance signals and domain-persistence analytics, rather than relying solely on user reports or copyright notices. Consumer-protection agencies could incorporate cross-domain clustering and refund-policy validation into routine e-commerce audits, while payment processors and domain registrars could be required to suspend merchants repeatedly associated with coordinated counterfeit activity. These measures would reposition freebooting from a niche copyright concern to a broader issue of digital market integrity and consumer safety.

Operationally, the findings point toward a viable integration pathway for platform-scale provenance enforcement. The components of the pipeline can be batched or streamed depending on latency constraints: 1) frame-level embeddings can be extracted during video upload or ad review, using GPU-accelerated batching to reduce compute costs; 2) audio fingerprints can be generated in streaming mode with constant-time updates per audio segment; and 3) the multimodal fusion classifier can operate either in near-real-time (<50 ms per asset on a single GPU) or in asynchronous backfill for long-tail provenance verification. Hashing and embedding layers scale linearly with video length, and approximate nearest-neighbor search via FAISS or ScaNN allows fast matching against large provenance databases (10^7–10^8 assets) at predictable computational cost. As such, integration into existing trust-and-safety pipelines would require minimal architectural change: the fusion classifier could be appended to the existing perceptual-hash checks used by most platforms, providing a “second layer” triggered only when hash confidence falls below a threshold, thereby controlling costs.

Ethical and legal considerations played a central role in the design of the study. All data were collected from publicly visible sponsored advertisements and publicly posted creator content; no private or restricted data were accessed. Creator privacy was safeguarded through the removal of usernames, handles, and personal identifiers in all stored datasets and reported outputs. Potentially illegal content—including counterfeit product listings and fraudulent storefronts—was archived in accordance with institutional data-handling guidelines, stored securely, and not redistributed. The study underwent review by the authors’ institutional ethics board (IRB-equivalent), which determined that the research qualified as minimal-risk observational work involving publicly available information. Because the findings revealed possible large-scale asset misuse and ongoing consumer harm, a coordinated responsible-disclosure process was initiated. Summary reports describing the counterfeit networks, freebooted assets, and associated domains were provided to TikTok’s integrity and IP-enforcement teams, as well as to House of CB’s brand-protection unit. Notifications included only domain-level evidence and never creator-identifying information. No investigative actions, takedowns, or enforcement decisions were triggered directly by this research; any subsequent moderation or domain removal that occurred during or after the study window was carried out independently by the respective platforms. The research team performed no adversarial probing, account infiltration, or purchasing beyond standard consumer interactions, and no attempt was made to trigger enforcement activities deliberately.

From a practical standpoint, the results underscore the limitations of current platform-level content moderation. Single-channel detection is insufficient against adversarially modified assets, while the lack of cross-platform provenance enables identical freebooted material to circulate unimpeded between TikTok, Instagram, and other environments (Amerini et al., 2025; Al-Tabakhi et al., 2024; Manchekar et al., 2024). The threat model documented here suggests concrete mitigations. For visual attacks such as animated borders and frame-in-frame layouts, detectors should incorporate spatially invariant embeddings and boundary-aware segmentation models. For TTS-based audio evasion, platforms could integrate mel-spectrogram-level anomaly detection and hybrid fingerprinting tuned for prosody-agnostic matching. For heavy filters and color grading, contrastive learning approaches that normalize style variations show promise. Adversarial training with synthetic perturbations—cropping, speed shifts, overlays, synthetic voices—could further harden each modality. Multimodal provenance should incorporate cross-channel consistency scoring so that evasion in one modality does not invalidate detection in others.

Taken together, these findings point toward the need for a product roadmap in which platforms adopt multimodal deduplication at upload, continuous cross-domain clustering to identify coordinated advertisers, and shared provenance databases that allow content to be recognized across TikTok, Instagram, Facebook, Snapchat, and short-video platforms yet to emerge. Upload-time multimodal deduplication would reduce the influx of freebooted material before it begins circulating, while cross-domain clustering would allow trust-and-safety teams to identify advertiser networks rather than individual ads, reducing whack-a-mole enforcement cycles. The economic infrastructure enabling counterfeit campaigns also requires attention. Payment processors and fulfillment intermediaries are central chokepoints in the counterfeit economy; integrating provenance signals into merchant verification workflows could prevent serial shell vendors from reappearing under new domains. Similarly, domain registrars and hosting providers could be required to incorporate basic identity disclosure or escrow verification for merchants repeatedly associated with counterfeit-linked ad clusters. Such measures fall within the broader remit of fintech compliance and anti-fraud programs and would reduce the incentives that currently reward rapid domain churn. From a policy perspective, the study points to a regulatory gap between intellectual property enforcement and consumer protection. While copyright takedowns address the interests of creators, the harms to consumers—non-delivery, low-quality products, fraudulent refund policies—remain under-regulated. Integrating freebooting into discussions of e-commerce fraud and online advertising transparency would better capture the socio-technical risks identified here. Regulators could prioritize requirements for advertiser verification, auditable refund mechanisms, and inter-platform cooperation on provenance metadata. In addition, consumer-protection authorities could incorporate multimodal provenance indicators into advertising disclosures, allowing end users to understand whether the creative used in an ad originated with the advertiser or was appropriated.

An often-overlooked dimension of freebooting is its impact on the creators whose content is appropriated. The results of this study show that influencers’ videos and images are repeatedly repurposed across dozens of counterfeit campaigns, frequently without attribution and sometimes with deceptive recontextualization (e.g., implying endorsement of low-quality or fraudulent products). This practice undermines the influencer’s brand equity, as their likeness and cultural capital are exploited to sell goods they neither created nor support. Such exploitation also introduces reputational risks. Consumers encountering freebooted ads may incorrectly assume that influencers are directly affiliated with the counterfeit brand, leading to erosion of trust when purchased products fail to meet expectations. For influencers whose livelihoods depend on authenticity and credibility, repeated association with dupes or scams poses long-term damage to audience relationships. In addition, freebooting strips influencers of economic value. Instead of benefiting from monetization through legitimate brand partnerships, creators are excluded from the revenue streams generated by counterfeit campaigns that leverage their content at scale. This constitutes not only a violation of intellectual property but also a redistribution of income from legitimate creative labor to fraudulent enterprises.

From a protective standpoint, the findings suggest that influencers should be more actively integrated into platform-level provenance systems. Watermarking, proactive fingerprinting of releases, and improved takedown APIs would enable creators to safeguard their assets before they are widely exploited. Furthermore, policy interventions should extend beyond copyright enforcement to explicitly recognize and compensate for the reputational and economic harms inflicted on influencers by freebooting practices. Although our study focused on TikTok and on fashion dupes, many structural mechanisms generalize across sectors. The socio-technical pipeline—asset theft, adversarial manipulation, algorithmic amplification, and counterfeit monetization—is not unique to apparel. However, reliance on visual similarity makes fashion particularly susceptible, and the generalizability of specific failure modes (e.g., TTS audio evasion or boundary overlays) may vary in verticals such as electronics, cosmetics, or health products. Future research is required to assess platform-level applicability beyond the TikTok ad ecosystem, especially in contexts with different ad formats, moderation systems, or regulatory requirements.

In sum, this study reframes freebooting as an adversarial strategy with multi-layered technical, economic, and social dimensions. It highlights both the ingenuity of counterfeit actors in exploiting socio-technical infrastructures and the insufficiency of current platform countermeasures. By situating freebooting within a broader framework of parasitic monetization and adversarial distribution, we provide a conceptual and methodological basis for future research on platform integrity, counterfeit economies, and the design of resilient detection systems. Although the present analysis focused on TikTok and on a single high-visibility fashion product category, the underlying socio-technical mechanisms identified here are not restricted to this vertical. The pipeline of asset theft, adversarial transformation, algorithmic amplification, and counterfeit monetization reflects structural incentives present across most short-video ecosystems, including Instagram Reels, YouTube Shorts, and Snapchat Spotlight. However, some findings are platform-specific and should not be overgeneralized. TikTok’s recommendation architecture, creative rotation patterns, and advertiser onboarding rules may produce diffusion dynamics that differ from platforms with heavier upfront verification or stricter media-library checks. Likewise, fashion dupes rely heavily on visual similarity, which may not translate directly to product categories where text overlays, technical specifications, or unboxing footage dominate (e.g., electronics, cosmetics, wellness products). Therefore, while the *process model* uncovered in this study is broadly extensible, the *quantitative rates*—such as TTFR, cluster density, fulfillment failure rates, or audio-fingerprint degradation—should be interpreted as characteristic of this specific TikTok-driven dupe ecosystem rather than universal constants. Future research using cross-platform, cross-category datasets will be necessary to determine the extent to which these dynamics generalize to other sectors with different media norms and adversarial incentives.

Despite the strong performance of the multimodal fusion model, several limitations of the detection pipeline warrant explicit acknowledgment. First, the threat model evaluated in this study—while broader than those typically used in platform-native systems—does not exhaust the full space of adversarial manipulations. We did not test attacks such as multi-layered compositing, deepfake-style facial re-synthesis, cross-modal desynchronization, or model-targeted adversarial perturbations engineered specifically to degrade embedding layers. These represent plausible next-generation evasion strategies and could further reduce recall if deployed at scale. Second, the pipeline does not incorporate cross-platform provenance tracking, meaning that the same assets circulating on Instagram Reels, YouTube Shorts, or Snapchat remain outside the detection graph. This creates blind spots in lineage reconstruction and limits the system’s ability to detect coordinated campaigns that span multiple platforms. Third, while quantitative robustness diagnostics provide estimates of false-positive and false-negative rates under controlled edits, real-world behavior may differ. False negatives—particularly under aggressive cropping, TTS substitution, or multi-layer audio mixing—would lead to undercounting of freebooted assets, thereby making our estimates conservative. Conversely, isolated false positives, though rare in validation tests, could overstate the size of particular reuse clusters or misattribute provenance in ambiguous borderline cases. These risks were mitigated through conservative thresholds, manual adjudication of uncertain matches, and exclusion of all non-unanimous provenance labels, but they nonetheless impose interpretive limits on the precision of diffusion metrics and cluster boundaries. Future work should extend the pipeline with adversarial training, platform-spanning fingerprint indices, and domain-adapted fusion models to reduce these residual vulnerabilities.

## Data Availability

The datasets presented in this study can be found in online repositories. The names of the repository/repositories and accession number(s) can be found at: DOI: 10.17632/2vbk4fcrsz.1.
